# The Role of Interleukin-1 Receptor-Associated Kinases in Vogt-Koyanagi-Harada Disease

**DOI:** 10.1371/journal.pone.0093214

**Published:** 2014-04-01

**Authors:** Min Sun, Peizeng Yang, Liping Du, Yan Yang, Jian Ye

**Affiliations:** 1 Department of Ophthalmology, Research Institute of Surgery & Daping Hospital, Third Military Medical University, Chongqing, P.R. China; 2 The First Affiliated Hospital, Chongqing Medical University, Chongqing Key Laboratory of Ophthalmology, Chongqing Eye Institute, Chongqing, P. R. China; Oregon Health & Science University, United States of America

## Abstract

**Purpose:**

To explore whether IRAK1 and IRAK4 are involved in the pathogenesis of Vogt-Koyanagi-Harada (VKH) disease.

**Methods:**

Thirty-nine VKH patients and thirty-two healthy controls were included in this study. The mRNA levels of IRAK1 and IRAK4 from active VKH patients, inactive VKH patients, and normal controls in peripheral blood mononuclear cells (PBMCs) were detected using real-time quantitative PCR. CD4^+^T cells were purified from PBMCs obtained from active VKH patients and normal controls. The effect of IRAK1/4 inhibition on CD4^+^T cell proliferation following stimulation with IL-18 or IL-1β was measured using a modified MTT assay. CD4^+^T cell expression of IFN-γ and IL-17 were detected by flow cytometry (FCM) and enzyme-linked immunosorbent assay (ELISA). The effect of IRAK1/4 inhibition on NF-κB, STAT1, and STAT3 activation was detected by FCM.

**Results:**

The mRNA levels of IRAK1 and IRAK4 were both significantly increased in active VKH patients compared to inactive VKH patients and healthy controls. No difference in the IRAK1 or IRAK4 mRNA level could be detected between inactive patients and healthy controls. After incubation with IRAK1/4 inhibitor, the proliferation of CD4^+^T cells was inhibited both in the active VKH patients and in the healthy controls. IRAK1/4 inhibition was also associated with a decreased expression of IFN-γ and IL-17. Phosphorylation of NF-κB, STAT1, and STAT3 in CD4^+^T from healthy controls was significantly decreased after inhibition of IRAK1/4.

**Conclusions:**

High mRNA levels of IRAK1 and IRAK4 correlated with VKH disease activity. IRAK1 and IRAK4 play a role in the activation and proliferation of CD4^+^T cells and the higher expression observed in VKH may contribute to the pathogenesis of this blinding condition.

## Introduction

Interleukin-1 receptor-associated kinases (IRAKs) are a unique family of death domain containing protein kinases that play a key role in the signaling cascades of two receptor families, toll-like receptors (TLRs) and interleukin-1 receptors (IL-1Rs). There are four mammalian members of the IRAK family: IRAK1, IRAK2, IRAK3 (IRAKM), and IRAK4. Although IRAKs are categorized as serine/threonine protein kinases and all contain a kinase-like domain, only IRAK1 and IRAK4 exhibit kinase activity [1], [2], [3]. The MyD88-dependent pathway, utilized by all TLRs except TLR3, signals via IRAK1 and IRAK4, which then associate with TRAF6, leading to the activation of transcription factors such as early phase NF-κB and AP-1, finally leading to the secretion of inflammatory cytokines, such as TNF-α, IL-1, and IL-6 [4], [5], [6].

It has been reported that mice deficient for IRAK4 are severely impaired in their cellular responses to IL-1, IL-18, and most TLR ligands, sharing an overlapping phenotype with IRAK4-deficient human patients [7]. However, although IRAK4-deficient mice display broad susceptibility to viral and bacterial infections, IRAK4-deficient human patients exhibit a narrow infectious phenotype, limited primarily to pyogenic bacterial infections at an early age [8], [9]. IRAK1 deficiency in humans has not been well described. It has been reported that IRAK1 may be a risk gene with a critical role in the pathogenesis of systemic lupus erythematosus (SLE) [10]. Gene polymorphisms (rs3027898, rs1059702) of IRAK1 were shown to be associated with SLE in a Chinese Han population[11]. IRAK1-deficient mice were impaired in their ability to develop experimental autoimmune encephalomyelitis (EAE) [12].

As mentioned above, the role of IRAKs in autoimmune disease has been reported for SLE and animal models of autoimmune encephalitis. The role of IRAKs in the pathogenesis of inflammatory eye disease has however not yet been investigated and was therefore the purpose of the study described here. We chose to study the role of IRAKs in a well-defined uveitis entity that is relatively frequently encountered in our uveitis clinic, namely Vogt-Koyanagi-Harada (VKH) disease. VKH is a multisystem autoimmune disorder directed against melanocyte antigens that mainly affects the pigmented tissues in the eye and the auditory, integumentary, and central nervous systems. Bilateral granulomatous panuveitis is a hallmark of VKH disease. It frequently results in severely decreased vision or even blindness if not treated properly [13], [14], [15]. It has been demonstrated that elevated levels of IL-17 and IFN-γ are associated with disease activity in patients with uveitis, including entities such as VKH disease [16]. Recent as yet unpublished studies from our group have shown that an enhanced expression of TLRs is associated with VKH providing a further basis to study downstream effector mechanisms such as IRAK in the development of this disease. In the present study we therefore investigated the expression and function of IRAKs in Vogt-Koyanagi-Harada Disease. Our results showed an increased expression of IRAK1 and IRAK4 in VKH patients with active uveitis. Further functional experiments were performed to provide an explanation for the role of IRAK1 and IRAK4 in the pathogenesis of this disease.

## Materials and Methods

### Patients and controls

During the study we included a total number of thirty-nine patients with VKH disease (23 men and 16 women), with an average age of 41.1 years, and 32 healthy individuals (18 men and 14 women), with an average age of 39.7 years. The diagnosis of VKH disease was made according to the diagnostic criteria revised for VKH disease by an international committee on nomenclature [17]. Twenty-four patients had active uveitis, as evidenced by diffuse bilateral choroiditis in association with exudative retinal detachment during the first uveitis attack or by mutton fat keratic precipitates, cells in the anterior chamber, and sunset glow fundus in VKH patients with recurrent episodes. The systemic findings included headache (79.5%), tinnitus (69.2%), dysacusis (41.0%), poliosis (33.3%), alopecia (41.0%), and vitiligo (20.5%). The patients included in the study had not used immunosuppressive agents for at least 1 week or used only a low dosage of corticosteroids (<20 mg/d) before blood sampling. Fifteen VKH patients showed no active intraocular inflammation (inactive uveitis stage) for at least 3 months after treatment with prednisone alone or combined with either chlorambucil or cyclosporin A for more than 1 year. Blood samples were obtained from these inactive patients at least 3 months after termination of all medications. This study was approved by the Ethics Committee of the Third Affiliated Hospital of the Third Military Medical University. All procedures complied with the Declaration of Helsinki and written consent was obtained from all patients with VKH disease and controls. The ethics committee approved this consent procedure.

### RNA preparation and real-time quantitative PCR

Peripheral blood mononuclear cells (PBMCs) were isolated using Ficoll-Hypaque density gradient centrifugation (Ficoll-Hypaque; TBDScience, Tianjin, China). Total RNA was extracted from PBMC with TRIzol (Invitrogen, Carlsbad, CA) according to the manufacturer's instructions. The first-strand cDNA was synthesized using the Reagent Kit (TaKaRa, Dalian, China). The primers and probes for real-time PCR of IRAK1 were purchased from QuantiFast Probe of Qiagen. The sense and antisense primers of IRAK4 used in this experiment were: sense: 5′TGATGGAGATGACCTCTGCT3′ and antisense: 5′GGTGGAGTACCATCCAAGCAA3′. Real-time quantitative PCR was performed on an AB7500 Fast System (Applied Biosystems). During the course of the project we first tested IRAK1 expression and later also included IRAK4 in a different set of patients and controls.

### Purification of CD4^+^T and culture

Peripheral CD4^+^T cells were purified from PBMCs by magnetic-assisted cell sorting (MACS) using a human CD4^+^T cell isolation kit according to the manufacturer's instructions (Miltenyi Biotec, Palo Alto, CA). The purity of isolated CD4^+^T cells, identified by FCM, was more than 95%. IRAK-1/4 inhibitor was obtained from Sigma-Aldrich (St. Louis, MO; catalog number: I5409) and was dissolved in dimethyl sulfoxide (DMSO) at 50 μM as a stock solution and stored at −20°C. CD4^+^T cells were resuspended at 1×10^6^ cells/ml in medium RPMI 1640 (Gibco, Invitrogen, Carlsbad, CA) containing L-glutamine (2 mM), penicillin/streptomycin (100 U/ml), and 10% fetal calf serum. CD4^+^T cells, activated with anti-CD3 (0.5 μg/ml) and anti-CD28 antibodies (0.1 μg/ml), were cultured with IRAK inhibitor (50 nM Sigma) or DMSO in the presence of recombinant IL-18 (100 ng/ml; R&D Systems, Minneapolis, MN) or recombinant IL-1β (20 ng/ml; PeproTech, London, U.K.) for 3 days. IL-18 was used to polarize the cells towards a Th1 subset [18], [19] whereas IL-1β was used to polarize the cells towards a Th17 subset [20].

### Flow cytometry

To detect the expression of intracellular cytokines in CD4^+^T cells, the cells were stimulated with 100 ng/ml phorbol 12-myristate 13-acetate (PMA) and 1 μg/mlionomycin (Sigma-Aldrich) for 5 hours. During the final 4 hours, 10 μg/ml Brefeldin A (Sigma-Aldrich) was added to the cultured CD4^+^T cells. The stimulated cells were washed, permeabilized, fixed, and subsequently stained with APC-conjugated IFN-γ mAbs and PE-conjugated IL-17 mAbs (eBioscience, San Diego, CA).

To detect the activities of transcription factors in CD4^+^T cells, the CD4^+^T cells were cultured with anti-CD3 antibodies (0.5 μg/ml) and anti-CD28 antibodies (0.1 μg/ml) for 30 minutes. Then, the stimulated cells were washed, permeabilized, fixed, and stained with PE-conjugated pSTAT3 mAbs, PerCP-Cy5.5 conjugated pSTAT1 mAbs, or Alexa Fluor 488 conjugated pNF-κB p65 mAbs, respectively (BD Bioscience, San Diego, CA).

Flow cytometry analysis was performed on a FACS Aria cytometer (BD Biosciences, San Diego, CA) and analyzed with FlowJo sofware (Tree Star, Inc., San Carlos, CA). Mean fluorescent intensity (MFI) was expressed as the geometric mean channel fluorescence minus the appropriate isotype control.

### ELISA

After 72 hours, the supernatants of cultured CD4^+^T cells were collected and stored at −80°C until the cytokine measurement. The levels of IL-17 and IFN-γ were measured using Duoset ELISA development kits (R&D Systems, Minneapolis, MN).

### Proliferation assay

Proliferation of CD4^+^T cells was induced by culturing the cells with IL-18 or IL-1β in the presence of anti-CD3 and anti-CD28 antibodies for 3 days. The effect of IRAKs on proliferation was investigated by adding IRAK inhibitor (50 nM) or the diluent DMSO to the culture system. The number of cells was quantitated by a modified MTT assay whereby, 10 μl WST-8 (2-(2-methoxy-4-nitrophenyl)-3-(4-nitrophenyl)-5-(2, 4-disu lfophenyl)-2H-tetrazolium, monosodium salt) was added to the culture system and incubated for the last 3 hours. The absorbance was determined at 450 nm using an ELISA reader (BIO-TEK Instruments). The experiment was repeated at least three times.

### Statistical analysis

Data were expressed as the mean ± standard deviation (SD). Groups were compared with paired samples T test, and a p value below 0.05 was taken as a statistically significant difference. The analysis was performed using SPSS software (SPSS version 13.0; SPSS, Chicago, IL)

## Results

### IRAK1 and IRAK4 mRNA level from PBMCs is increased in active VKH patients

**Figure 1:**
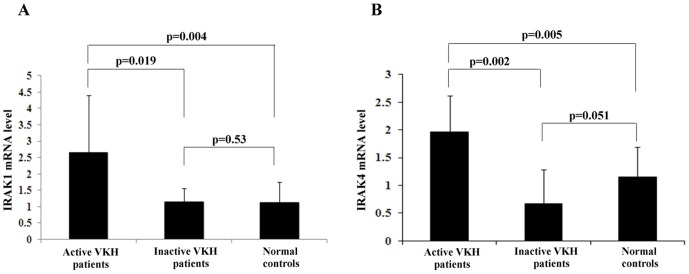
The mRAN levels of IRAK1 and IRAK4 in active VKH patients, inactive VKH patients and normal controls. **A**. The mRNA level in PBMCs of IRAK1 of active VKH patients (n = 18), inactive VKH patients (n = 12), and normal subjects (n = 20) was detected by real-time quantitative PCR. **B**. The mRNA level in PBMCs of IRAK4 of active VKH patients (n = 12), inactive VKH patients (n = 8), and normal subjects (n = 10) was detected by real-time quantitative PCR.

The mRNA level of IRAK1 and IRAK4 in PBMCs was determined by real-time PCR. The results showed that the expression of IRAK1was significantly higher than that in inactive VKH patients (p = 0.019) and normal controls (p = 0.004). There was no significant difference in the IRAK1 mRNA level between inactive VKH patients and the healthy controls (p = 0.53) (Figure 1A).

The expression of IRAK4 in active patients was also higher than that observed in inactive patients (p = 0.002) and normal controls (p = 0.005). There was no significant difference of the IRAK4 mRNA level in PBMCs between inactive VKH and the healthy controls (p = 0.051) (Figure 1B).

### Effect of IRAK1/4 inhibition on the proliferation of CD4^+^T cells

**Figure 2:**
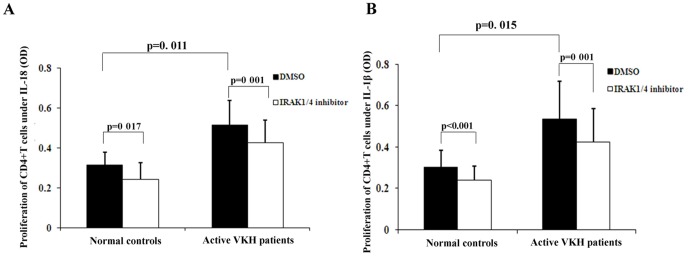
The inhibitory effect of IRAK1/4 inhibitor on the proliferation of CD4^+^T cells purified from PBMCs obtained from active VKH patients (n = 10) and normal controls (n = 11). Cells were cultured with IRAK1/4 inhibitor (50 nM) in the presence of IL-18 (100 ng/ml) or IL-1β (20 ng/ml) for 3 days. Anti-CD3 mAb (0.5 μg/ml) and anti-CD28 mAb (0.1 μg/ml) were added to the culture system at the same time. Proliferation of cultured CD4^+^T cells was tested by a modified MTT assay. Results are expressed as means ± SD.

CD4^+^T cells were purified from PBMCs obtained from both active patients and healthy controls. The CD4^+^T cells were cultured in the presence of IRAK1/4 inhibitor (50 nM) in the presence of rIL-18 (100 ng/ml) or rIL-1β (20 ng/ml) for 3 days whereafter we measured its effect on cell proliferation. Anti-CD3 and anti-CD28 antibodies were added to the system to mimic antigen presentation and IL-18 or IL-1β was added to polarize the T cells towards the Th1 or Th17 subset, respectively. In the presence of rIL-18, the proliferation of CD4^+^T cells from active VKH patients was significantly higher than that from normal controls (p = 0.011). After co-culturing with rIL-1β, the proliferation of CD4^+^T cells from active VKH patients was also significantly higher than that from normal controls (p = 0.015). The proliferation of CD4^+^T cells from both active patients and normal controls was decreased significantly after cells were cultured in the presence of IRAK1/4 inhibitor (Figure 2).

### Effect of IRAK1/4 inhibitor on the expression of IFN-γ and IL-17

**Figure 3:**
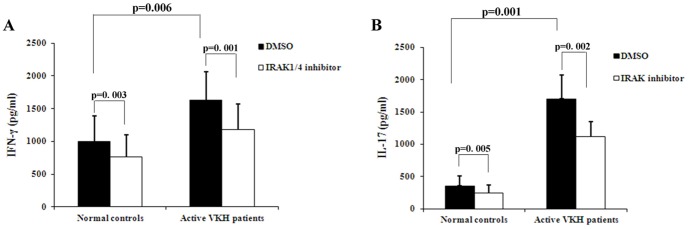
Inhibitory effect of IRAK1/4 inhibitor on the secretion of IFN-γ and IL-17 by CD4^+^T cells from active VKH patients (n = 10) and normal controls (n = 11). Cells were cultured with or without IRAK1/4 inhibitor in the presence of anti-CD3 and anti-CD28 antibodies for 3 days. Meanwhile, IL-18 or IL-1β was also added to the cultures. The level of IFN-γ or IL-17 was measured by ELISA. A. In the presence of IL-18, IFN-γ production by CD4^+^T cells from patients with active VKH disease (n = 10) and normal controls (n = 11). Results are expressed as means ± SD. B. In the presence of IL-1β, IL-17 production by CD4^+^T cells was measured in patients with active VKH disease (n = 10) and normal controls (n = 11). Results are expressed as means ± SD.

To study the effect of IRAK1/4 on the expression of IFN-γ by CD4^+^T cells we performed the following experiment. Antigen stimulation of Th1 cells was mimicked by using a cocktail of anti-CD3, anti-CD28 antibodies and rIL-18. The results showed that the expression of IFN-γ in active VKH patients (1631.1±431.0 pg/ml) was significantly higher than that in normal controls (994.1±390.0 pg/ml, p = 0.006). After co-culturing with IRAK1/4 inhibitor, the level of IFN-γ was decreased both in active VKH patients (1178.8±391.6 pg/ml, p = 0.001) and in healthy controls (761.6±338.6 pg/ml, p = 0.003) (Figure 3A).

Similar experiments were performed to address the role of IRAK1/4 on Th17 cells. For this purpose CD4^+^T cells were stimulated with anti-CD3, anti-CD28 antibodies, and rIL-1β. A similar result was also observed in this experiment. The level of IL-17 in active patients (1704.1±368.3 pg/ml) was obviously higher than that in healthy control (354.4±157.7 pg/ml). The IRAK1/4 inhibitor suppressed the secretion of IL-17 both in active VKH patients (1118.1±230.8 pg/ml, p = 0.002) and in normal controls (249.5±115.4 pg/ml, p = 0.005) (Figure 3B).

### Influence of IRAK1/4 inhibitor on the frequencies of IFN-γ and IL-17 expressing CD4^+^T cells

**Figure 4:**
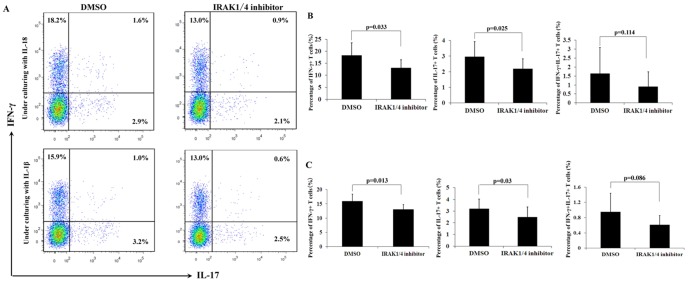
The effect of IRAK1/4 inhibitor on the expansion of Th1 cells and Th17 cells. Purified CD4^+^T cells from normal controls (n = 8) were cultured with or without IRAK1/4 inhibitor for 3 days in the presence of wither IL-18 or IL-1β. The frequency of Th1 and Th17 cells was analyzed by FCM. **A**. A representative patient with data near the mean of each group in B and C. **B**. The results represent the percentages of IFN-γ^+^, IL-17^+^, and IL-17^+^IFN-γ^+^ cells among the CD4^+^T cells in the presence of IL-18. Results are expressed as means ± SD. **C**. The results represent the percentages of IFN-γ^+^,IL-17^+^, and IL-17^+^IFN-γ^+^ cells among the CD4^+^T cells in the presence of IL-1β. Results are expressed as means ± SD.

The results mentioned above demonstrated that the IRAK1/4 inhibitor could inhibit the secretion of both IFN-γ and IL-17 from CD4^+^T cells in active VKH patients and healthy controls. In a following set of experiments, we investigated whether IRAK1/4 inhibitor could influence the frequencies of CD4^+^IFN-γ^+^T cells, CD4^+^IL-17^+^T cells, and CD4^+^IFN-γ^+^IL-17^+^T cells in healthy controls. Purified CD4^+^T cells were treated with or without IRAK1/4 inhibitor to evaluate its influence on the frequencies of these cells. In the presence of rIL-18 or rIL-1β, the addition of IRAK1/4 inhibitor significantly suppressed the frequency of CD4^+^IFN-γ^+^T cells (rIL-18: from 18.2% to 13.0%, p = 0.033; rIL-1β: from 15.9% to 13.0%, p = 0.013). Under the same conditions, IRAK1/4 inhibitor also suppressed the frequency of CD4^+^IL-17^+^T cells (rIL-18: from 2.9% to 2.1%, p = 0.025; rIL-1β: from 3.2% to 2.5%, p = 0.03). However, IRAK1/4 inhibitor had no effect on the frequency of CD4^+^IFN-γ^+^IL-17^+^T cells in the presence of rIL-18 or rIL-1β (rIL-18: from 1.6% to 0.9%, p = 0.114; rIL-1β: from 1.0% to 0.6%, p = 0.086) (Figure 4).

### Effect of IRAK1/4 inhibitor on the activation of NF-κB

**Figure 5:**
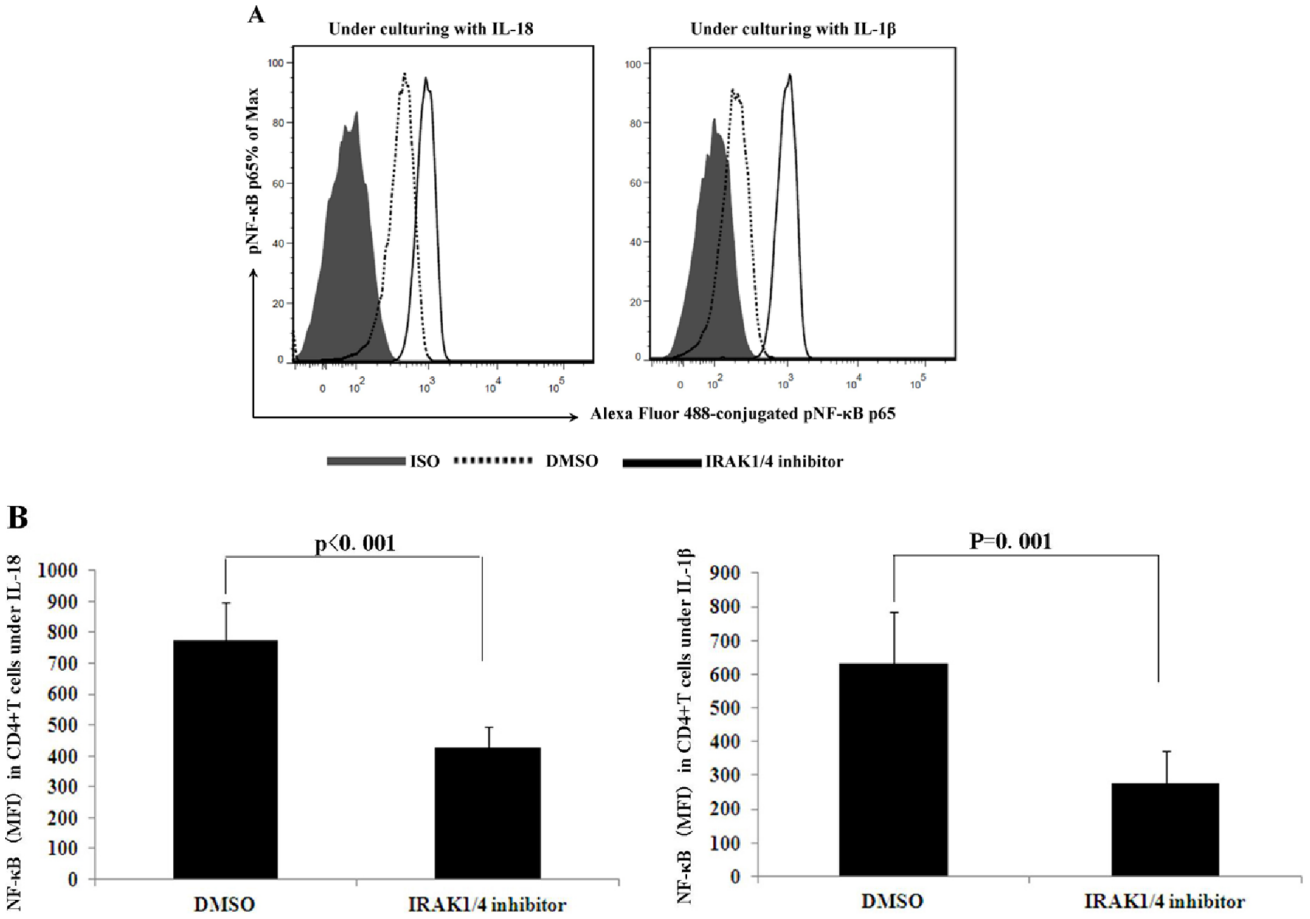
The effect of IRAK1/4 inhibitor on the phosphorylation of NF-κB. Purified CD4^+^T cells from normal controls (n = 6) were cultured with or without IRAK1/4 inhibitor in the presence of anti-CD3 and anti-CD28 antibodies for 30 minutes. **A**. A representative example with data near the mean of each group in group B. **B**. The results represent the mean fluorescence intensity of phosphorylation NF-κB expression in the CD4^+^T cells. Results are expressed as means ± SD.

It has been demonstrated that NF-κB activation is associated with inflammation in a wide array of immune mediated diseases and animal models [21]. NF-κB is responsible for the transcription of genes encoding pro-inflammatory cytokines and chemokines [22]. To investigate the role of IRAK1/4 on the activity of NF-κB, we stimulated CD4^+^T cells with anti-CD3 and anti-CD28 antibodies in the presence of an inhibitor. Co-culturing with rIL-18 or rIL-1β, IRAK1/4 inhibition resulted in a decrease in the mean fluorescence intensity (MFI) of phosphorylation NF-κB from 774 to 426 (p<0.001) and from 632 to 278 (p = 0.001), respectively (Figure 5).

### IRAK1/4 inhibitor inhibited the activity of STAT1 and STAT3

**Figure 6:**
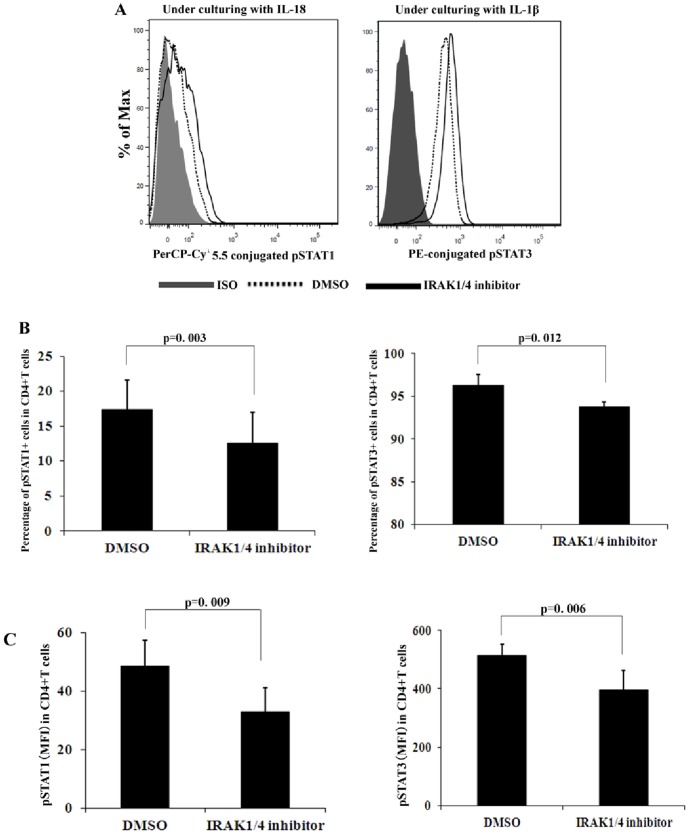
The effect of IRAK1/4 inhibitor on the activities of STAT1 and STAT3. Purified CD4^+^T cells from normal controls (n = 6) were cultured with or without IRAK1/4 inhibitor in the presence of anti-CD3 and anti-CD28 antibodies for 30 minutes. The phosphorylation of STAT1 and STAT3 were analyzed by FCM. **A**. A representative patient with data near the mean of each group in groups B and C. **B**. The results represent the percentage of pSTAT1 and pSTAT3 among the CD4^+^T cells and (**C**) the intensity of pSTAT1 and pSTAT3 in the CD4^+^T cells. Results are expressed as means ± SD.

Among the STATs, the STAT1 and STAT3 protein have emerged as important determinants of whether naïve T cells differentiate into the Th1 or Th17 cell lineage [23]. Given the importance of these subsets in the pathogenesis of VKH, we determined the activation of STAT1 and STAT3 under Th1 or Th17 polarizing conditions in healthy controls (Figure 6). In the presence of rIL-18, the addition of IRAK1/4 inhibitor significantly suppressed the frequency of phosphorylated STAT1 from 17.4% to 12.5% (p = 0.003). Additionally, IRAK1/4 inhibitor reduced the MFI of STAT1 from 48.6 to 32.9 (p = 0.009). In the presence of rIL-1β, the addition of IRAK1/4 inhibitor significantly suppressed the frequency of phosphorylated STAT3 expressing cells from 96.3% to 93.8% (p = 0.012) and reduced the fluorescence intensity from 514 to 395 (p = 0.006), respectively. IRAK1/4 inhibition did not lead to significant effects on STAT4 activation (data not shown).

## Discussion

In the present study, we investigated the expression of IRAK1 and IRAK4 and the possible roles of these two kinases in the pathogenesis of VKH disease. The results showed that the mRNA levels of both IRAK1 and IRAK4 were significantly increased in PBMCs obtained from patients with active VKH disease. In vitro experiments showed that IRAK1/4 inhibitor could inhibit the proliferation of CD4^+^T cells, as well as the expression of both IFN-γ and IL-17. Experiments with IRAK1/4 inhibitor further showed that IRAK1/4 exerts its function by activating NF-κB, STAT1, and STAT3.

Our study is the first to report on the role of IRAKs in clinical uveitis. An earlier study showed that IRAK-M (now named IRAK-3) was induced in the mouse eye following intraocular injection of endotoxin [24]. The limitation of the present study is that the IRAK1 and IRAK4 protein levels in VKH patients and normal controls were not detected. It is not clear why the systemic mRNA expression of IRAK1 and IRAK4 is increased in our patients. Gene polymorphisms may alter the expression of IRAKs and further studies are needed to show whether this may offer an explanation for our findings. Although the borderline IRAK4 mRNA level in normal controls was higher than that observed in inactive VKH patients, the results did not reach statistical significance. Further studies using a larger sample size should be done to clarify whether this trend can be confirmed. As yet no explanation can be given for this phenomenon.

Previous studies from our group have shown the importance of Th1 and Th17 pathways in the development of VKH disease [25], [26]. In this study, we investigated whether the IRAKs can influence the secretion of IFN-γ and IL-17, which are considered the representative cytokines for Th1 and Th17 cells, respectively. Our results showed that both the frequencies and the expression of IFN-γ and IL-17 were suppressed after incubating CD4^+^T cells with IRAK1/4 inhibitor in active VKH patients and normal controls. The suppressive effects we observed were modest and one should take into account that a large variety of cytokines and their signal transduction pathways are involved in an ongoing inflammatory response. Additive effects of these various pathways may all lead towards the same pro-inflammatory direction finally leading to overt clinical manifestations. Our data should thus not be interpreted as that IRAKs are the crucial controlling elements, but that they are only part of a complex machinery causing inflammation.

We explored the mechanisms through which the IRAK1/4 pathway might exert its suppressive role in the inflammatory response by using PBMCs from normal subjects. NF-κB was first discovered and characterized 25 years ago as a key regulator of inducible gene expression in the immune system[27]. The activation of IRAK4 and IRAK1 results in the activation of transcription factors, including early phase NF-κB and AP-1[6]. We showed that incubation of CD4^+^T cells with IRAK1/4 inhibitor combined with a stimulation using anti-CD3 antibodies and anti-CD28 antibodies, resulted in a significant decrease of the NF-κB phosphorylation. Others have demonstrated that delphinidin could inhibit the phosphorylation of IRAK1 in IL-1β-induced activation of NF-κB in human chondrocytes [28], which illustrated that phosphorylation of IRAK1 was associated with the activation of NF-κB. In combination with our results, we speculate that IRAK1/4 inhibitor can suppress inflammation by inhibiting the activity of NF-κB.

Along with early phase activation of NF-κB, CD4^+^T cells can proliferate and expand towards different effector T cells, such as Th1 and Th17 cells following an encounter with various antigens. As demonstrated in our study, IRAK1/4 inhibitor could inhibit the proliferation of CD4^+^T cells under both Th1 and Th17 polarization conditions. Previous studies demonstrated that commitment to Th1 and Th17 lineages requires STAT1- and STAT3-dependent mechanisms, which facilitate and/or induce IFN-γ and IL-17 expression [23], [29]. Therefore, we further verified whether IRAK1/4 inhibitor exerted its suppressive function through influencing the activities of STAT1 and STAT3. The in vitro experiments demonstrated that IRAK1/4 inhibitor suppressed the phosphorylation of STAT1 and STAT3. It has also been demonstrated that commitment to the Th1 lineage requires STAT4-dependent mechanisms. In our study, we could not detect an influence of IRAK1/4 inhibitor on the activity of STAT4 [30]. The reasons for these discrepancies are not yet clear and deserve further study.

In conclusion, our study provides evidence that IRAK1 and IRAK4 are involved in the development of active VKH disease. Higher IRAK expression in this disease may lead to enhanced proliferation of CD4^+^T cells, and an increased secretion of IFN-γ and IL-17. Activation of IRAKs follows the signal transduction pathways involving phosphorylation of NF-κB, STAT1, and STAT3. Further studies are needed to explore whether IRAK1/4 may be used as a target to treat this blinding condition.
